# Thyroid metastasis from lung carcinoid detected by ^68^Ga-DOTATOC PET/CT

**DOI:** 10.1007/s12020-021-02706-0

**Published:** 2021-04-05

**Authors:** Domenico Albano, Raffaele Giubbini, Francesco Bertagna

**Affiliations:** Nuclear Medicine, University of Brescia and ASST Spedali Civili Brescia, Brescia, Italy

A 62-year-old woman with a history of atypical lung carcinoid with mediastinal lymph nodes metastases previously treated with left pneumectomy and hilum-mediastinal lymph node dissection (pT3N2M0; 2 mitoses/10HPF and 25% Ki-67 index), underwent a restaging 68gallium DOTATOC positron emission tomography/computed tomography (^68^Ga-DOTATOC PET/CT). PET/CT images were acquired 60 min after the intra-venous injection of 198 MBq of ^68^Ga-DOTATOC on a Discovery 690 tomograph (GE; Milwaukee, Wis; 64-slice CT, 80 mA, 120 kV; 2.5 min/bed; 256 × 256 matrix, 60-cm field of view).

**Fig. 1:**
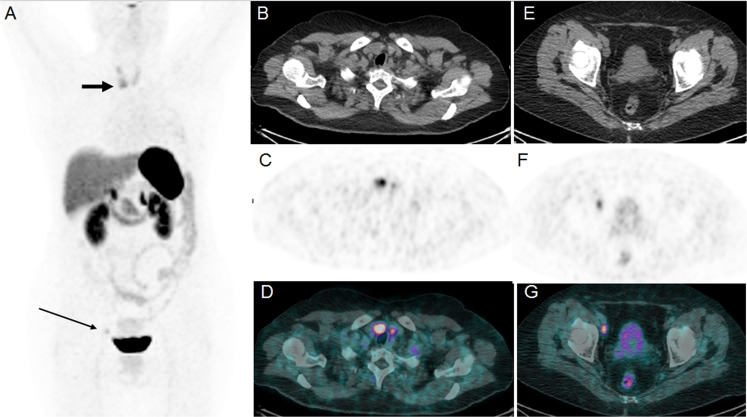
Maximum intensity projection (MIP) showing a mild increased DOTATOC uptake on right iliac lymph node (slim arrow) and focal high uptake on the right thyroid lobe (thick arrow) (**A**). Axial noncontrast enhanced CT showing a nodule of 18 mm on the lower part of the right thyroid lobe (**B**), axial PET (**C**) and PET/CT fused images with the increased uptake corresponding to the nodule detected by CT (**D**). Axial CT (**E**), PET (**F**) and PET/CT fused (**G**) images showing a right external iliac lymph node

^68^Ga-DOTATOC PET/CT images revealed an increased radiotracer uptake corresponding to an external iliac lymph node and a focal uptake on the lower part of the right lobe of the thyroid (Fig. 1). Thyroid function, serum calcitonin and carcinoembryonic antigen were measured and were in the reference range. Subsequent ultrasound ultrasound detected a solid, hypoechoic with irregular margin single thyroid nodule of the left thyroid lobe and fine-needle aspiration cytology (FNAC) was performed and showed a cytological and immunohistochemical pattern consistent of thyroid metastasis from lung carcinoid. The immunohistochemical analyses revealed a strong immunoreactivity to Chromogranin A and synaptophysin. Thus, the finding was considered as thyroid metastasis of neuroendocrine nature, probably of lung carcinoid.

Metastases to thyroid gland are very uncommon despite the high vascularization of this organ. Breast, lung, stomach and renal cell carcinoma are the most common primary site of localization of thyroid metastases. Instead, thyroid metastases from neuroendocrine cancer are even more rare and may be difficult to differentiate from medullary thyroid carcinoma.

A physiological diffuse faint thyroid uptake with ^68^Ga-DOTA peptides is common, due to the somatostatin receptors expression in normal thyroid tissue, while focal increased radiotracer uptake are usually corresponding to differentiated thyroid carcinoma or thyroid nodules [1, 2]. Diffuse thyroid ^68^Ga-DOTA uptake is usually due to thyroiditis nature [1].

The finding of a thyroid metastasis from lung carcinoid detected by ^68^Ga-DOTATOC PET/CT is exceptional with only one case in literature [3]. In conclusion, this report underlines the importance of further investigations need of incidental findings in the thyroid at ^68^Ga-DOTA PET/CT, especially in patients with anamnesis positive for neuroendocrine lesions, potentially pivotal for an adequate patient management.
